# Accuracy of Serological Testing for the Diagnosis of Prevalent Neurocysticercosis in Outpatients with Epilepsy, Eastern Cape Province, South Africa

**DOI:** 10.1371/journal.pntd.0000562

**Published:** 2009-12-08

**Authors:** Humberto Foyaca-Sibat, Linda D. Cowan, Hélène Carabin, Irene Targonska, Mushtaq A. Anwary, Gilberto Serrano-Ocaña, Rosina C. Krecek, A. Lee Willingham

**Affiliations:** 1 Walter Sisulo University, Mthata, South Africa; 2 Department of Biostatistics and Epidemiology, College of Public Health, University of Oklahoma Health Sciences Center, Oklahoma City, Oklahoma, United States; 3 Dora Nginza Hospital, Port Elizabeth, South Africa; 4 Ross University School of Veterinary Medicine, Basseterre, Saint Kitts and Nevis; 5 Department of Zoology, University of Johannesburg, Auckland Park Campus, Auckland Park, South Africa; 6 WHO/FAO Collaborating Center for Parasitic Zoonoses, Danish Center for Experimental Parasitology, Faculty of Life Sciences, University of Copenhagen, Frederiksberg, Denmark; University of Maryland School of Medicine, United States of America

## Abstract

**Background:**

Few studies have estimated prevalence of neurocysticercosis (NCC) among persons with epilepsy in sub-Saharan Africa. While the limitations of serological testing in identification of NCC are well known, the characteristics of persons who are misdiagnosed based on serology have not been explored. The first objective of this pilot study was to estimate the prevalence of NCC in epilepsy outpatients from an area of South Africa endemic for cysticercosis. The second objective was to estimate the accuracy of serological testing in detecting NCC in these outpatients and characterize sources of disagreement between serology and neuroimaging.

**Methodology/Principal Findings:**

All out-patients aged 5 or older attending the epilepsy clinic of St. Elizabeth's Hospital in Lusikisiki, Eastern Cape Province, between July 2004 and April 2005 were invited to participate. Epidemiological data were collected by local study staff using a standardized questionnaire. Blood samples were tested by ELISA for antibody and antigen for *Taenia solium*. Four randomly chosen, consenting participants were transported each week to Mthatha for brain CT scan. The proportion of persons with epilepsy attending St. Elizabeth clinic with CT-confirmed NCC was 37% (95% CI: 27%–48%). Using CT as the gold standard, the sensitivity and specificity of antibody testing for identifying NCC were 54.5% (36.4%–71.9%) and 69.2% (52.4%–83.0%), respectively. Sensitivity improved to 78.6% (49.2%–95.3%) for those with active lesions. Sensitivity and specificity of antigen testing were considerably poorer. Compared to false negatives, true positives more often had active lesions. False positives were more likely to keep pigs and to have seizure onset within the past year than were true negatives.

**Conclusions/Significance:**

The prevalence of NCC in South African outpatients with epilepsy is similar to that observed in other countries where cysticercosis is prevalent. Errors in classification of NCC using serology alone may reflect the natural history of NCC.

## Author Summary

Epilepsy is a significant contributor to morbidity world-wide in persons of all ages. Little is known, however, about its causes. In the developing world, parasitic infections of the brain, in particular *Taenia solium* neurocysticercosis (NCC) are thought to be important factors. Determining whether or not there is infection in the brain is difficult since to be certain, specialized imaging studies, such as CT scans, are required. These are expensive and not widely available. In addition, they are not appropriate for use in large, population-based studies. Thus, blood tests for evidence of infection with *T. solium* are often done instead to estimate the presence of NCC. In this study's population of persons with epilepsy being seen at a hospital out-patient clinic in South Africa, 37% had CT evidence of NCC, a percentage similar to that reported in other developing countries. The study also found that blood tests were not generally useful compared to CT for correctly identifying those persons who did or did not have NCC, and thus, they cannot be relied upon for field studies of NCC.

## Introduction

Neurocysticercosis (NCC) results when the central nervous system (CNS) is invaded by the larval stage of *Taenia solium*, a zoonotic cestode transmitted between humans and pigs. Humans infected with the adult tapeworm, a disease called taeniasis, contaminate the environment with the parasites' eggs contained in the proglotids that are shed in their feces. Pigs become infected with the eggs and the larvae migrate to several tissues, including muscles. Humans acquire taeniasis when eating insufficiently cooked, infected pork meat. In areas where sanitation and personal hygiene are poor, humans can also become infected with the eggs present in feces of people infected with taeniasis. Eggs develop to larvae, enter the CNS and can lodge in the parenchyma, subarachnoid space, or the ventricular system of the brain and are sometimes seen in the eye [1]. Since each proglotid contains about 40,000 eggs and infected humans may shed a few proglotids per day, a single carrier can be responsible for the infection of multiple individuals and pigs in a community [1],[2].

The manifestations of NCC are protean, but approximately 80% to 90% of persons with symptomatic NCC experience seizures at some time in the course of their illness [3],[4], making this manifestation a reasonable starting point for identifying NCC cases in populations at risk. Acute symptomatic seizures (seizures occurring only in the presence of an acute central nervous system (CNS) process) in NCC are thought to result from inflammatory responses in the brain in response to a degenerating cyst [5]. Calcified intraparenchymal cysts may serve as a focus for remote symptomatic seizures as late sequelae of NCC [5], but there is not always concordance between the location of calcified cysts and a seizure focus identified by electroencephalogram [6].

The proportion of persons with acute symptomatic seizures associated with NCC who go on to develop epilepsy (recurrent, unprovoked seizures) is unknown, and determining the proportion of seizures attributable to NCC is problematic in the absence of repeated serological testing and concurrent diagnostic neuroimaging. In spite of these difficulties, NCC is considered to be the leading preventable cause of epilepsy in developing counties [7]–[9]. Some of the studies used to support this contention have not discriminated between acute symptomatic seizures and epilepsy or have counted as NCC cases persons with seizures and positive serology for *T. solium* (for example, Garcia et al., 1993 [10]; Nsengiyumva et al. 2003 [11]). In the absence of neuroimaging evidence of brain lesions compatible with NCC, it is not possible to determine whether infection with *T. solium* may be the cause of the epilepsy. This is especially of concern in areas endemic for cysticercosis where many people may be exposed to the eggs of the parasite, including exposure after the onset of epilepsy. The relation between number, location and stage of NCC lesions and the presence and type of clinical manifestations further complicates the discrimination between acute symptomatic seizures and epilepsy in the field [10], [12]–[15].

Alternatives to serology for identifying lesions of NCC in the brain are computerized tomography (CT) or magnetic resonance imaging (MRI) of the brain. The results of the imaging combined with epidemiological and serological results have been suggested by an international panel of experts for the diagnosis of NCC [16]. Reliance on neuroimaging studies for the diagnosis of NCC is not generally feasible, however, especially in developing countries where the disease is likely to be most prevalent and CT and MRI are often not available. In addition, it is not a reasonable method for case identification in community-based field studies of the epidemiology of NCC. Development of alternative, valid strategies for identifying persons with NCC would greatly facilitate such studies.

The first objective of this pilot study was to estimate the proportion with NCC in persons attending an outpatient clinic for epilepsy in an area of South Africa endemic for cysticercosis. A second objective was to estimate the accuracy of antibody and antigen serological testing in detecting cases of NCC in patients with epilepsy.

## Methods

The design of this study is cross-sectional.

### Selection of Participants

Persons eligible for the pilot study were out-patients who were attending the epilepsy clinic of St. Elizabeth's Hospital in Lusikisiki, Eastern Cape Province, South Africa. These patients were either referred for seizures from a rural clinic or presented themselves directly to the hospital for seizure diagnosis and care. All individuals aged 5 years or older seen at St. Elizabeth's Hospital between July 2004 and April 2005 with possible new onset epilepsy (incident cases) and those returning for continuing care of epilepsy (prevalent cases) were invited to participate. Clinical and epidemiological data were collected by local study staff for all eligible consenting patients. Attempts were also made to obtain blood samples from all participants in order to test for the presence of antibody and antigen for *T. solium*.

### Data Collection

A standardized questionnaire, adapted from Preux [9] and developed by the Cysticercosis Working Group of Eastern and Southern Africa (CWGESA), was administered in either English or isi-Xhosa. Isi-Xhosa is spoken by the vast majority (95%) of the black population in the Eastern Cape Province [17]. Translation from English to isi-Xhosa and back translation to English was done by two independent local residents who were fluent in both languages. The interview included questions designed to screen for epilepsy as well as information about potential risk factors for and knowledge of cysticercosis and taeniasis, pig-keeping practices of the household, and questions on their expenditures for medical care for seizures. (Copies of the questionnaire are available on request.) Mothers or primary care takers completed the interview for children.

Clinical data for each participant, including the diagnosis of epilepsy and the types of seizure manifestations, were provided by the local co-investigator (G.S.-O.). The International League Against Epilepsy (ILAE) classification of seizure types was used to designate primary type of seizure based on descriptions of seizure manifestations in the clinical data [18],[19]. For these analyses, epilepsy was defined as a clinical diagnosis of seizures in combination with self-report from the questionnaire of ever having had more than one seizure. At least two seizures separated by at least 24 hours had to have occurred for a patient to be diagnosed with epilepsy or recurrent unprovoked seizures. An incident case of epilepsy was defined based on questionnaire response as occurrence of first seizure within the past year.

### Serological Testing

Serological testing for *T. solium* antibody and antigen was offered to all participants. Serological testing for the presence of antibody to *T. solium* was done using the ELISA method with purified antigens (RIDASCREEN® *Taenia solium* IgG commercial diagnosis kit (R-Biopharm, Darmstadt, Germany)) [20]. Blood serum samples were collected and transported from Lusikisiki to Mthatha where serum was removed, aliquoted and frozen. One frozen serum aliquot was transported to East London to detect the presence of antibodies in the serum using an ELISA test. The second serum aliquot was transported to the Medical Research Council in Cape Town to detect the presence of antigens in the serum using an ELISA.

The test used to detect antigens was the B158/B60 monoclonal antibody-based ELISA developed by Dorny et al., 2000 [21]. For this Ag-ELISA the calculation of the cut off is based on the results of eight negative controls. The optical density (OD) of each serum sample is compared with the mean OD of a series of eight samples from non-infected individuals at a probability level of P<0.001 to determine the result in the test [22]. The calculation is based on Student's t-test.

In some analyses, the results of the antigen-ELISA and antibody-ELISA were considered together to define a positive or negative result. If both measures were positive, the combined measure was considered positive; if both were negative, the combined measure was negative. A positive result to either test was considered positive, even if the other result was missing. Similarly, a negative result to either test was considered negative, even if the other result was missing. These assumptions were considered reasonable since among patients with both antigen and antibody results available, 95% (38/40) of those with a negative antibody result also had a negative antigen result. Only one person had data missing for both antigen and antibodies.

### Selection of Participants for the CT-Scan Examination

Each week, four randomly chosen patients with clinically diagnosed seizure disorder and who gave written consent were transported for CT scan of the brain (Toshiba Asteion TSX-021A) to the Nelson Mandela Academic Hospital (NMAH), a teaching hospital affiliated with the Walter Sisulu University for Technology and Science in Mthatha. The transportation, per diem and cost of the CT were covered by the research project and did not incur any costs to the patients. When estimating the proportion of cases associated with NCC, it is essential that seizure patients be sampled randomly for CT in order to avoid persons suspected of having NCC being preferentially referred. The random sampling was done by first randomly selecting a day of the week (Monday through Friday). The first four eligible patients at St. Elizabeth's seen on the randomly chosen day were referred to the NMAH. If there were not four eligible patients on that day, the remaining number required to reach a total of four were chosen from the first eligible patients seen the following day. If the random day was Friday and there were not four eligible patients on that day, then the remainder were selected on the following Monday. CT scans were done with and without radiographic contrast material for all referred patients unless contrast was contraindicated (e.g., allergy to contrast media, pregnancy). Scans were read by radiologists (IT, MAA) at the NMAH as part of usual clinical care. Readings were done without knowledge of serological results. For this pilot study, results of the CT scans were used as the “gold standard” for classification of NCC. We recognized that imaging is not a true gold standard for the diagnosis of NCC but there were no other imaging facility available to the patients in Mthatha. It is reasonable, however, to assume that the CT-scan will be a much better test for identifying lesions in the brain and a serological test. Using the CT report results, cases were classified as “NCC” (either evidence of visible cysticerci, colloidal cysts or presence of cerebral calcifications with a specific CT diagnosis of NCC) or “not NCC” (no diagnosis of NCC and no evidence of cerebral calcifications of any kind). Serology results were not used in making these designations. Persons with CT scans interpreted as “cerebral calcifications” but without mention of NCC and who had no evidence of active cysticerci were included in one set of estimates of the proportion of epilepsy associated with NCC but were excluded from analyses of NCC in order to reduce the possibility of misclassification.

### Data Management and Analysis

All interview data were independently entered in an Access® database by two individuals. Questions for which there was a disagreement of entries of more than 5% were reviewed and corrected. Questions with disagreements of less than 5% were considered acceptable. Sixty percent of all entries had a mismatch of 2% or less. Prevalence proportion ratios, sensitivity, specificity and predictive values were estimated. The 95 percent confidence intervals (95%CI) of the field-based diagnosis of NCC were calculated assuming a binomial distribution and using the exact Clopper-Pearson method [23]. Data were examined for confounding and interaction for all covariates, but since neither was present, univariate results are reported. All analyses were conducted using SAS version 9.1 (SAS Institute, Inc., Cary, North Carolina).

### Consent Procedures

Two signed consent forms were used in this study. The first consent form asked participants for their agreement to be interviewed and have a blood sample drawn for the serological analysis. A second consent was used for those patients selected at random to have a CT-scan in Mthatha. This project was approved by the Institutional Review Boards of the University of Oklahoma Health Sciences Center, the Walter Sisulo University, and St. Elizabeth Hospital.

## Results

A total of 296 individuals with suspected epilepsy were seen at the outpatient clinic in St. Elizabeth of whom 281 were diagnosed with at least one seizure according to the clinical report. Socio-demographic and clinical characteristics as well as serological results from these patients are shown in Table 1. Ages ranged from 5 to 76 years with 31 (11.0%) cases being children (i.e., <16 years of age). The seroprevalence of antibodies and antigens to the larval stage of *T. solium* were 32.6% (95%CI: 27.1%–38.5%) and 7.9% (95%CI: 4.5%–12.8%), respectively. Generalized seizures were reported slightly more often than partial seizures. Of the generalized seizures, tonic-clonic were most often reported, and the most commonly observed partial seizures were either complex partial seizures with motor manifestations (n = 79, 28.1%) or partial seizures secondarily generalized (n = 40, 14.3%). A total of 244 patients (86.8%, 95%CI: 82.3%–90.6%) met our definition of epilepsy. Among those with epilepsy, the onset of seizures was within the past year in 28.9% (95%CI: 23.0%–35.4%) of cases, while another 31.2% (95%CI: 25.1%–37.8%) had been having seizures for 10 years or longer at the time of this study.

**Table 1:** Demographic, clinical, and serological characteristics of 281 patients with seizures, St-Elizabeth Hospital Outpatient Clinic, July 2004–April 2005.

Characteristic	Category	N	%	95% CI†
Number of participants		281		
Age range		5–76 yrs.		
Age group	Children (<16 yrs)	31	11.0	7.6–15.3
Gender	Female	163	58.0	52.0–63.8
Self-reported frequency of seizures	Single seizure	17	6.1	3.6–9.5
	More than one seizure	244	86.8	82.3–90.6
	No answer	20	7.1	4.4–10.8
Primary seizure type	Partial seizures	131	46.6	40.7–52.6
	Generalized seizures	150	53.4	47.4–59.3
Reported duration of seizures+	≤1 year (incident)	63	28.9	23.0–35.4
	2–4 years	50	22.9	17.5–29.1
	5–9 years	37	17.0	12.2–22.6
	≥10 years	68	31.2	25.1–37.8
*T. solium* antibody*	Positive	89	32.6	27.1–38.5
*T. solium* antigen*	Positive	15	7.9	4.5–12.8
Brain CT	Yes	111	39.5	

**†:** 95% confidence interval.

*: of those tested (antibody testing, n = 273; antigen testing, n = 188).

**+:** of those who answered duration question and had available seizure frequency data (n = 218 of 281).

Since the referral of patients for CT was done randomly, the characteristics of those with and without CT are expected to be similar. There were more patients sent for CT for whom seizure frequency was unknown (13.5% vs. 2.9%) and more children aged less than 16 (16.3% vs 7.2%), but no other statistically significant differences were observed in demographic, seizure type or duration, or serological results between those who were or were not referred for CT. The distributions of demographic and clinical characteristics of those referred as compared to those not referred for a CT scan are given in Table S1.

A total of 92 patients who met the study's definition of epilepsy received a CT scan. Of those, 34 (37.0%, 95%CI: 27.1%–47.7%) had a CT diagnosis of NCC; 20 (21.7%; 95%CI: 13.8%–31.6%) of these showed calcification with a diagnosis of NCC and 14 (15.2%; 95%CI: 8.6%–24.2%) had either visible cysticerci or colloidal lesions (subsequently referred to as “active” NCC). In these two groups, the proportions with new-onset seizures were 31.6% (95%CI: 12.6%–56.6%) and 46.2% (95%CI: 19.2%–74.9%), respectively. If persons with epilepsy who had CT evidence of cerebral calcifications not diagnosed as NCC (n = 19) were included as possible cases of NCC, the proportion of epilepsy associated with NCC would be 43/92 or nearly 47%. These 19 uncertain cases were excluded from further analysis of NCC as these lesions may be due to other cerebral infections such as tuberculosis. Some cases of NCC may have been missed by this approach, which would under-estimate the proportion of seizure cases associated with NCC. Excluding them has the benefit, however, of reducing the chances of misclassification within the NCC group, which increases the validity of the comparisons between NCC and non-NCC epilepsy cases.

Characteristics of those with calcified NCC lesions, ‘active’ NCC, and those with no CT abnormalities are summarized in Table 2. Those with ‘active’ lesions were less often female and more often seropositive than those with calcified lesions or those with no CT abnormalities.

**Table 2:** Comparison of characteristics in epilepsy patients in those with a CT diagnosis of NCC calcifications, those with “active” NCC and those with no CT abnormalities.

Characteristics	Category	NCC Dx** (n = 20)	“Active” NCC (n = 14)	No abnormality (n = 39)
Age group	≥16 years	15 (75%)	11 (78.6%)	32 (82.1%)
Gender	Female	12 (60%)	6 (42.9%)	24 (61.5%)
Seizure type	Partial	6 (30%)	7 (50%)	18 (46.2%)
	Generalized	14 (70%)	7 (50%)	21 (53.8%)
Reported duration of seizures	≤1 year (incident)	6 (31.6%)	6 (46.2%)	7 (19.4%)
	2–4 years	3 (15.8%)	2 (15.4%)	10 (27.8%)
	5–9 years	5 (26.3%)	2 (15.4%)	6 (16.7%)
	≥10 years	5 (26.3%)	3 (23.1%)	13 (36.1%)
*T. solium* antibody	Positive	7/19 (36.8%)	11/14 (78.6%)	12/39 (30.8%)
*T. solium* antigen	Positive	2/13 (15.4%)	2/10 (20%)	2/22 (9.1%)

See text for complete definition of the result groupings.

**: Dx = diagnosis.

Prevalence proportion ratios for selected characteristics were calculated comparing those with: 1) any definite NCC diagnosis to those with no CT abnormality, and 2) those with ‘active’ NCC to those with no abnormality (Table 3). The small number of cases in each group is reflected in the wide 95% CI. When compared to those with epilepsy who had no CT abnormalities, the proportion of incident cases of epilepsy tended to be higher in those with NCC (PPR = 1.97, 95%CI: 0.87–4.42) and even higher in those with ‘active’ NCC (PPR = 2.39, 95%CI: 0.97–5.89). The prevalence of a positive antibody test was higher in those with NCC (PPR = 1.77, 95%CI: 1.01–3.12), especially in those with ‘active’ NCC (PPR = 2.55, 95%CI: 1.48–4.40) as compared to those without NCC. Compared to those with calcified NCC lesions, the prevalence of seropositivity for antibody to *T. solium* was 2.55 times higher (95%CI: 1.48–4.40) in those with ‘active’ NCC.

**Table 3:** Prevalence proportion ratios (and 95% confidence intervals) comparing characteristics of those with any NCC (n = 34) to those with no CT abnormality (n = 39) and of those with “active” NCC (n = 14) to those with no CT abnormality.

Characteristic	All NCC vs no CT abnormality	“Active” NCC vs no CT abnormality
Age<16 yrs	1.31 (0.74–1.18)	1.19 (0.36–3.99)
Male Gender	1.22 (0.72–2.09)	1.49 (0.81–2.71)
Partial Seizures	0.83 (0.48–1.43)	1.08 (0.58–2.02)
Incident epilepsy*	1.97 (0.87–4.42)	2.39 (0.97–5.89)
Ab positive	**1.77 (1.01–3.12)** **	**2.55 (1.48–4.40)** ¶
Ag positive	1.91 (0.39–9.41)§	2.20 (0.36–13.47)±

*: Seizure onset within past year (n = 68).

**: Based on 72 observations.

**¶:** Based on 53 observations.

**§:** Based on 39 observations.

**±:** Based on 32 observations.

The validity of serological testing for antibodies or antigens as diagnostic tools for NCC in persons with epilepsy was estimated separately (Table 4). The predictive value for NCC of a positive antibody test was 60.0% (95%CI: 40.6%–77.3%). Hence, 40% (95%CI: 22.7%–59.4%) of those testing positive to the antibody ELISA but without NCC would be falsely attributed to NCC (false positives) based on serology alone. When this analysis was confined to those who had ‘active’ NCC, the sensitivity of serology in epilepsy patients was higher (78.6%, 95%CI: 49.2%–95.3%). However, since the prevalence of ‘active’ NCC is lower than that of all NCC, the predictive value of a positive antibody test in these patients was reduced to only 47.8% (95%CI: 26.8%–69.4%), therefore leading to 52.2% false positives.

**Table 4:** Estimated validity of field-based criteria (seropositive to antibodies and antigens of cysticercosis separately) for the diagnosis of all NCC and “active” NCC in persons with epilepsy.

FIELD-BASED RESULT	CT SCAN RESULT		
	All NCC	“Active” NCC	NO CT Abnormality
**Antibody**			
Antibody positive	18	11	12
Antibody negative	15	3	27
Field diagnosis validity (%)	Sensitivity: 54.5 (36.4–71.9)	Sensitivity: 78.6 (49.2–95.3)	Specificity: 69.2 (52.4–83.0)
Positive predictive value (%)	60.0 (40.6–77.3)	47.8 (26.8–69.4)	
Negative predictive value (%)	64.3 (48.0–78.4)	90.0 (73.5–97.9)	
**Antigen**			
Antigen positive	4	2	2
Antigen negative	19	8	20
Field diagnosis validity (%)	Sensitivity: 17.4 (5.0–38.8)	Sensitivity: 20.0 (2.5–55.6)	Specificity: 90.9 (70.8–98.9)
Positive predictive value (%)	66.7 (22.2–95.7)	50.0 (6.8–93.2)	
Negative predictive value (%)	51.2 (34.8–67.6)	71.4 (51.3–86.8)	

The predictive value of a positive antigen test was similar to that for antibody at 66.7% (95%CI: 22.2%–95.7%) but the sensitivity was very poor at 17.4% (95%CI: 5.0%–38.8%). When the validity of antigen testing was examined only among those with ‘active’ NCC lesions, the predictive value for a positive antigen test was 50.0% (95%CI: 6.8%–93.2%).

Serological studies might be more useful in identifying cases of NCC if only those with new onset (incident) seizures were assessed. Unfortunately, there were only 14 patients with new onset seizures who had a CT-scan and either antigen or antibody serology performed. Nonetheless, analysis in this small group suggested that being seropositive for *T. solium* may be a good field diagnostic tool for NCC among incident seizure cases (predictive value of positive serology = 80%, 95%CI: 44%–98%), although 40% (95%CI: 5%–85%) of those whose seizures are not related to NCC will be falsely categorized by serology as NCC.

A series of comparisons was made between those who were correctly and incorrectly classified as NCC by serology (antibody ELISA or antigen ELISA) in order to see whether there were characteristics that distinguished them. Serological true positives (CT positive for NCC and seropositive) and false negatives (CT positive for NCC but seronegative) as well as serologically true negatives (CT negative for NCC and seronegative) and false positive (CT negative for NCC and seropositive) are compared in Table 5. The small numbers in each group are reflected in the wide 95% CI. More true positives had ‘active’ lesions (PPR = 3.06, 95%CI: 1.04–8.97) than false negatives. Persons with epilepsy who are falsely identified as cases of NCC based on serology were more often present or past owners of pigs (PPR = 1.35, 95%CI: 1.08–1.69) and had a higher prevalence of incident seizures (PPR = 1.23, 95%CI: 1.02–1.47) compared to those correctly identified by serology as epilepsy not due to NCC.

**Table 5:** Distribution and associated prevalence proportion ratios (PPR) of demographic and clinical characteristics of 33 epilepsy patients with CT-diagnosed NCC lesions and 39 patients without CT-scan lesions of NCC according to serological field-based diagnosis (antigen or antibody ELISA positive).

Characteristic	Field-based classification					
	True positive (n = 18)	False negative (n = 15)	PPR* (95% CI)	True negative (n = 27)	False positive (n = 12)	PPR** (95% CI)
Age<16 yrs	5 (27.8%)	2 (13.3%)	2.08 (0.47–9.24)	4 (14.8%)	3 (25.0%)	1.69 (0.44–6.40)
Male gender	9 (50.0%)	6 (40.0%)	1.25 (0.58–2.71)	7 (25.9%)	5 (41.7%)	1.61 (0.67–4.26)
Generalized Seizure	11 (61.1%)	10 (66.7%)	0.92 (0.55–1.53)	11 (40.7%)	7 (58.3%)	1.43 (0.74–2.77)
Active NCC by CT	11 (61.1%)	3 (20.0%)	**3.06 (1.04–8.97)**	NA	NA	NA
Ever kept pigs	14 (82.4%)	11 (78.6%)	1.05 (0.74–1.49)	20 (74.1%)	12 (100%)	**1.35 (1.08–1.69)**
Reported duration of seizures≤1 year+	8 (47.1%)	4 (28.6%)	1.65 (0.62–4.34	22 (81.5%)	12 (100%)	**1.23 (1.02–1.47)**

*: PPR: prevalence proportion ratio calculated as % true positive with factor ÷ % false negative with factor).

**: PPR: prevalence proportion ratio calculated as % false positive with factor ÷ % true negative with factor).

**+:** Reported duration of seizures based on 31 observations for the true positive, false negative comparison.

## Discussion

The source population for this study included persons seeking medical care for symptomatic seizures (acute seizures) or recurrent asymptomatic seizures (epilepsy) at an outpatient clinic of St. Elizabeth Hospital, a rural hospital in Lusikisiki, Eastern Cape Province, South Africa. While the study includes consecutive, unselected epilepsy patients, this group does not represent a community-based sample of persons with epilepsy since only those seeking care are represented. The characteristics of these patients with respect to age distribution and seizure types were similar, however, to those observed in community-based studies of epilepsy conducted in sub-Saharan Africa [9],[11],[24].

Nearly one-third of epilepsy patients were seropositive to *T. solium* antibodies. This proportion is within the range of that reported from endemic countries of Sub-Saharan Africa using the antibody-ELISA test [25],[26].

When CT results are used to identify NCC, the proportion of epilepsy patients with evidence of NCC was 37%, which is higher than what would have been identified based on either ELISA test. This proportion is also comparable to many reports based on neuroimaging findings from other endemic regions [25],[27]. Only 15% of those with seizures had lesions with either a demonstrated scolex or that were described as colloidal or cystic; seizures in these patients may be more correctly classified as acute symptomatic seizures.

One of the aims of this pilot study was to estimate the validity of serological testing for *T. solium*, as a readily available classification tool in the field, in identifying persons with NCC among those with epilepsy or recurrent seizures. When using CT-scan results as the “gold standard”, only 60% of those seropositive to the antibody to *T. solium* truly had NCC and nearly one-third of those truly without NCC would be wrongly identified as NCC based on serology alone. Also, more than one-third of those seronegative to the antibodies of *T. solium* had NCC, and would thus not have been diagnosed using this field-based criterion. Some of the relatively poor sensitivity and specificity of serology in correctly identifying persons with NCC may be a result of the antibody ELISA test used in this study, which is based on purified antigens. However, in a study conducted using 67 sera and 53 CSF samples from confirmed cases of NCC in Chile, the sensitivity and specificity of the ELISA using a purified antigen was 97% and 98.3% for the serum samples, respectively [28]. We have used a commercially available kit which may have slightly different accuracy values. Other antibody ELISA tests using crude extract of the metacestodes have shown some limitations. In a double blind, head-to-head comparison in the same population in which only 6% of NCC cases had calcified lesions, EITB was more sensitive than antibody-ELISA based on crude extract of the metacestode in detecting antibody to *T. solium* (86% vs. 41% respectively) but approximately equal in specificity (93% v. 96%, respectively) [13]. In populations in which the proportion of NCC with calcified or single lesions is high, both the ELISA test using crude extract of the metacestode [13] or the EITB [29],[30] have significantly reduced sensitivity.

We found that antigen testing was very poor for identifying persons with NCC, even among those with active disease. This is likely due to the natural history of NCC in which the incubation period for CNS manifestations is not clearly understood but appears to be extremely variable; symptoms can occur many years after the primary infection [31]. While the breakdown of cysts in the brain and subsequent release of parasite antigen is thought to trigger an inflammatory response which results in acute CNS manifestations in NCC [5], antigens in the CNS may not be present at detectable levels in the serum. This would be contrary, however, to what has been demonstrated for antibodies detectable by EITB, in which samples from sera were more often positive than samples from cerebral spinal fluid [13]. Unfortunately, this pilot study was too small to examine the validity of positive serology for antigen in those with new onset seizures.

False negatives, i.e., those with NCC by CT but with negative serology, were less likely than true positives to have new onset seizures. Failure to identify these cases by antibody or antigen serology as possible NCC is compatible with the reported decline in detectable levels of antibody to *T. solium* over time [10],[32] and is a potential source of significant misclassification of NCC in all prevalence studies that use serology to detect antibodies, either with ELISA or EITB, to identify NCC in persons with epilepsy [33]. In addition, the performance of antibody-detection test (EITB) has been shown to be reduced among NCC cases showing single, small enhancing lesions [30]. False positives (no NCC by CT but seropositive) likely include persons whose epilepsy is from other causes who are seropositive for *T. solium* because they live in an endemic area. People who are positive to the presence of antibodies may have been infected in the past or may have been exposed to the cysts and developed sufficient antibodies to prevent the cysts from establishing in the body. People who are positive to the presence of antibodies or antigens may be infected with the cyst elsewhere in their body. These groups contribute to the over-estimation of the proportion of epilepsy due to NCC when only serological testing is used to identify cases.

In this study, neuroimaging by computerized tomography was used as the “gold standard” against which serology was compared; no MRI facilities were available in the study area. Misclassification of NCC based on CT would occur if the scan missed existing lesions (false negatives) or if lesions were misidentified as NCC (false positives). If present, such misclassification in the “gold standard” would impact the apparent performance of serology. The potential for false positives was reduced by excluding those with calcifications of uncertain nature and also by assessment of the validity of serology to the subset of cases with CT evidence of cysterci. This latter group is unlikely to be misclassified. CT and MRI are equally valid for parenchymal lesions, such as those in the present study [34],[35], while CT is considered superior to MRI for identifying calcified lesions, which represented 59% of NCC cases in the present study [15],[34]. Misclassification of serological results was reduced by requiring that results be definitely positive; those with intermediate results were not counted as seropositive.

The data available for the present study made it difficult to separate symptomatic seizures from epilepsy. We did include as epilepsy only those who self-reported having had more than one seizure episode and clearly, the duration of seizures was very long for many individuals, but the presence of some acute symptomatic seizures cannot be ruled out. It is interesting to note that the predictive value of positive serology for antibody to *T. solium* was highest in those with either visible cysteci or colloidal lesions on CT, suggesting that antibody testing might be useful for identifying acute symptomatic seizures, but not epilepsy.

The use of serological testing as a method for identifying cases of NCC in the field will continue to be inadequate because of the natural history of NCC. Although not fully understood because of the absence of cohort data, available cross-sectional and case-control information has helped to define some aspects of the disease which impact on the validity of any attempt to measure it in population settings. These characteristics include the evolving nature of the CNS lesions and how the location, stage and number of lesions may affect the clinical manifestations of NCC [5],[10],[12],[14],[15]; the unknown nature of the balance between intensity of infection and measurable antibody levels vs. CNS invasion; the duration of measurable antibody and antigen levels in relation to the presence of observable CNS involvement [10],[32]. Given these limitations, it is unlikely that even improved serology will alone be sufficient for the valid identification of persons with NCC.

## Supporting Information

Table S1 Comparison of those referred or not referred for CT scan.
(0.03 MB DOC)

Checklist S1 STROBE checklist.
(0.08 MB DOC)
