# Supplementary material for: Accuracy of Serological Testing for the Diagnosis of Prevalent Neurocysticercosis in Outpatients with Epilepsy, Eastern Cape Province, South Africa
Source: PLoS Negl Trop Dis. 2009 Dec 8;3(12):e562. doi: 10.1371/journal.pntd.0000562 (PMC2780704; doi:10.1371/journal.pntd.0000562)
Supplement: Table S1 — Comparison of those referred or not referred for CT scan. (0.03 MB DOC) [file pntd.0000562.s001.doc]

Appendix 1. Comparison of those Referred or not Referred for CT scan

| **Characteristic** | **CT (n=92)**  **N (%)** | **No CT (n=152)**  **N (%)** | **% difference (95% CI)** |
| --- | --- | --- | --- |
| Children (<16 yrs) | 15 (16.3) | 11 (7.2) | 9.1 (-2.2; 13.4) |
| Female | 55 (59.8) | 84 (55.3) | 4.5 (-6.4; 17.1) |
| Primary seizure type |  |  |  |
| Partial seizures | 45 (48.9) | 70 (46.1) | 2.8 (-7.1; 16.8) |
| Generalized seizures | 47 (50.5) | 82 (55.3) | -2.8 |
| Reported duration of seizures+  ≤ 1 year (incident)  2-4 years  5-9 years  ≥ 10 years | 21 (27%)  19 (20%)  13 (16%)  33 (37%) | 42 (35%)  31 (23%)  24 (17%)  35 (25%) | -7.4  -1.4  -3.1  11.9 |
| *T. solium* antibody positive* | 33/91 (36.3) | 44/147 (29.4) | 6.4 (-3.6; 19.3) |
| *T. solium* antigen positive* | 7/59 (11.9) | 8/102 (7.8) | 4.1 (-4.8; 13.0) |

+ of those who answered duration question (n=86 with CT and 132 without CT)

* Antibody positive excludes indeterminate result; antigen positive includes + and

++ but excludes traces
